# Editorial: Apoptosis Induction/Suppression: A Feasible Approach for Natural Products to Treatment of Diseases

**DOI:** 10.3389/fphar.2021.725496

**Published:** 2021-07-09

**Authors:** Wei Peng, Hong Zhang

**Affiliations:** ^1^ School of Pharmacy, Chengdu University of Traditional Chinese Medicine, Chengdu, China; ^2^ Institute of Interdisciplinary Integrative Medicine Research, Shanghai University of Traditional Chinese Medicine, Shanghai, China

**Keywords:** natural product, apoptosis, proliferation, cancer, diabetes

Apoptosis is recognized as the most important form of programmed cell death, which is involved in normal cell development, organ growth, and tissue homeostasis in multicellular organisms. Under normal conditions, there is a balance between cell death and proliferation every day in the human body, but the damage of this balance can cause some serious diseases.

There are two different case scenarios: 1) uncontrolled cell proliferation and insufficient cell apoptosis would result in cancers and autoimmune diseases; 2) excessive apoptosis in normal cells, in particular neural cells or cardiomyocytes, would lead to neurodegenerative diseases, such as Alzheimer’s disease and Parkinson’s disease, and ischemia injuries, such as myocardial infarction and stroke. Therefore, regulation of cell apoptosis is a useful strategy for the treatment of various diseases, especially cancers. Currently, increasing evidence has suggested that natural products derived from plants, animals, microorganisms, and minerals are beneficial for controlling some incurable and life-threatening diseases *via* regulating apoptosis, such as cancers, Alzheimer’s disease, Parkinson’s disease, stroke, etc.

The research topic “Apoptosis Induction/Suppression: A Feasible Approach for Natural Products to Treatment of Diseases” provide an academic platform to discuss the novel signal molecules for the apoptosis-related signal pathway, and how natural products can be used to treat several types of diseases through induction or suppression of apoptosis.

## Natural Products Induce Apoptosis in Tumors

Previous evidence suggested that the leading reasons for the development of cancers are closely related to the uncontrolled cell proliferation and insufficient apoptosis of cancer cells, therefore it’s no doubt that induction of apoptosis is an ideal strategy for treating and controlling different types of cancers. Tang et al. presented a review of the Tibetan herbal medicine for treating cancers by targeting apoptosis, and another review by Gan et al. summarized the plant polysaccharides which could be beneficial for controlling or treating different types of cancers by induction of apoptosis. In addition, Tian et al. gave a review regarding the anticancer effect of herbal medicine on colitis-associated cancer *via* induction of apoptosis. Besides, Lu et al. also reviewed the pharmacological effects of brucine, and found that this compound has promising antitumor effects against various cancers *via* apoptosis induction. Similarly, Zhang et al. indicated another known natural compound called matrine can also elicit apoptosis in different types of cancer cells. Using network pharmacology approach, Zhang et al. investigated the anti-lung cancer effect of active constituents from *Rheum palmatum* which is a known traditional herbal medicine widely used in China, and found that induction of apoptosis is the leading cause for the antitumor properties of this herbal medicine. Breast cancer is a life-threatening one in women with a high incidence rate, Saleem et al. investigated the anticancer effect of a natural monomer named Proscillaridin A, and the results suggested that this natural compound can induce apoptosis in breast cancer cells and the cell death is improved by blocking JNK-induced autophagy. Another paper by Peng et al. reported that the isoliquiritigenin derivative of 3′,4′,5′,4″-tetramethoxychalcone could treat the triple-negative breast cancer *via* inducing apoptosis. Xie et al. reported that alkaloids extracted from *Moringa oleifera* can inhibit prostate cancer cells growth related to apoptosis induction Yu et al. revealed that cantharidin induces apoptosis in acute myeloid leukemia cells by Nur77 related signaling pathway. Furthermore, Li et al. reported that eriodictyol inhibits the proliferation and metastasis of glioma cells by apoptosis induction via PI3K/Akt/NF-κB signaling pathway.

## Natural Products Inhibit Apoptosis in Other Diseases

In contrary to cancers, anti-apoptosis is beneficial for treating some other disorders, such as ischemia-reperfusion (I/R) injury related diseases, bone loss related diseases, etc. Tian et al. investigated the effect of a Chinese herbal formula named Sanqi oral solution on renal I/R injury, and found that this herbal formula can ameliorate the renal IR injury *via* reducing apoptosis and enhancing autophagy which is involved in the ERK/mTOR pathway. Another study by Zhang et al. reported that a natural monomer of Aucubin can attenuates liver I/R injury by inhibiting inflammatory response, oxidative stress, and apoptosis. In addition, Li et al. gave a review regarding role of the mammalian mitochondrial permeability transition pore (MPTP) in the neuronal apoptosis of ischemic stroke, and suggested that MPTP is a potential target of herbal medicines for stroke treatment by apoptosis inhibition.

In the present topic, some scientific evidence also showed that natural herbal medicine could be used to treat bone loss related diseases. Wei et al. reported that betulinic acid can inhibit bone loss in ovariectomized mice and suppress RANKL-associated osteoclastogenesis by inhibition of the MAPK and NFATc1 pathways. Similarly, Zhan et al. and Ai et al. reported, respectively that vindoline and theaflavin-3,3′-Digallate can also inhibit RANKL-induced osteoclastogenesis and prevent ovariectomy-induced bone loss in mice by suppressing MAPK activation and ROS production. Sun et al. studied the effect of pregnenolone on osteoclast *in vivo* and *in vitro*, and found that this natural compound can inhibit osteoclast differentiation, LPS-induced inflammatory bone destruction, and ovariectomy-induced bone loss in mice. Furthermore, Wu et al. suggested that salvianolic acid A possesses anti-osteoarthritis potentials in mice *via* inhibiting the apoptosis of mouse chondrocytes.

Interestingly, in our topic, some researchers reported that natural agents are also beneficial for improving some complications of diabetes. Wang et al. reported that scutellarin can inhibit apoptosis in the liver of STZ-induced T2DM rats and in cultured LO2 cells treated with homocysteine. In addition, Wu et al. reported hyperoside could ameliorate diabetic retinopathy related to inhibiting apoptosis in rat retinal vascular endothelial cells (RVECs) induced by high glucose. Xie et al. studied the protective effect of ginsenoside Re on RF/6A cells, and found that this compound could reduce high-glucose-induced RF/6A injury through apoptosis inhibition *via* regulation of PI3K/Akt and HIF-1α/VEGF signaling pathways.

Besides, in our present topic, Fang et al. reported quercetin can attenuate d-GaLN-induced L02 cell injury by inhibition of oxidative stress and mitochondrial apoptosis. Shen et al. found that the extract of Erhuang formula, a Chinese herbal prescription, can improve renal fibrosis in diabetic nephropathy rats through inhibition of fibroblast proliferation and decrease of the expression of inflammation and fibrosis related factors. Liu et al. studied the Chinese herbal medicine formula of *Sanhuang Xiexin* decoction, and found that this formula could reduce the endotoxemia induced apoptosis.

We believe that our present topic shows the latest scientific research on regulation of apoptosis for natural products to treat different types of diseases, including fundamental theory and experiments *in vitro* and *in vivo*. These investigations suggest that apoptosis induction/suppression might be beneficial in the control of various diseases, including tumors, ischemia-reperfusion (I/R) injury related diseases, bone loss related diseases, complications of diabetes, etc. However, in the present research, some other important apoptosis related diseases have not been mentioned, such as the neurodegenerative diseases (Alzheimer’s disease, Parkinson’s disease, and Huntington’s disease), autoimmune diseases (rheumatoid arthritis and lupus erythematosus), etc. Therefore, more investigations should be devoted in exploration of novel natural products with curative activities in neurodegenerative diseases, autoimmune diseases *via* apoptosis induction/suppression.

